# Criterion Validity of the “HRQOLISP-E”: A New Context-Specific Screening Tool for Poststroke Depression

**DOI:** 10.1155/2017/6515769

**Published:** 2017-10-11

**Authors:** Akin Ojagbemi, Mayowa Owolabi, Joshua Akinyemi, Bruce Ovbiagele

**Affiliations:** ^1^College of Medicine, University of Ibadan, Ibadan, Nigeria; ^2^Kwame Nkrumah University of Science and Technology, Kumasi, Ghana

## Abstract

**Objectives:**

The optimal tool for identifying postsroke depression (PSD) is yet to be identified. In the present study, we rely on the depression subscale of the Hospital Anxiety and Depression Scale (HADS-D) as a meaningful criterion to investigate the psychometric properties of the HRQOLISP-E, a new context-specific screening tool for PSD developed from a large cross-cultural sample.

**Methods:**

We assessed baseline data being collected as part of an intervention to improve one-year blood pressure control among recent (≤one month) stroke survivors. Depression was measured using the HADS-D and the HRQOLISP-E. We determined sensitivity, specificity, likelihood ratios, and posttest probability. The area under a receiver operator curve (AUC) and the most appropriate HRQOLISP-E cut-off were also determined using standard procedures.

**Results:**

Using data derived from 387 recent stroke survivors, the HRQOLISP-E showed high agreement with the HADS-D, sensitivity = 73.7%, specificity = 79.3%, and posterior test probability = 88% (95% CI = 84%–91%). The AUC was 0.81 (95% CI = 0.76–0.86). The HRQOLISP-E cut-off, corresponding to HADS-D score ≥ 8, was 20/21 (out of a total score of 30).

**Conclusions:**

Within limitations of using the HADS-D as a referent criterion, the present results provide justification for further development of the HRQOLISP-E as the first stroke-specific screening tool for depression.

## 1. Introduction

Stroke is a leading cause of death and disability globally [1], with depression being a key complication accounting for much of poststroke morbidity [2]. Though highly prevalent among stroke survivors, depression remains a neglected entity due to underrecognition and undertreatment [3]. Yet, timely and comprehensive treatment of poststroke depression (PSD) is important for effective management of stroke [4, 5]. As such, routine screening of stroke survivors for depression is increasingly recommended [6].

The recognition of PSD is complicated by somatic and cognitive symptoms which are common in stroke survivors regardless of emotional complications [7]. However, because currently available screening tools for PSD were originally designed for general psychiatric use [8], they include many symptoms which conflate their scores, thus limiting content validity for stroke. Consequently, it remains very difficult to identify the most appropriate screening tool for PSD. However, the depression subscale of the Hospital Anxiety and Depression Scale (HADS-D), though expensive to acquire, was found to be psychometrically adequate for PSD screening in the two available systematic reviews of all such tools [8, 9].

Using the method of factor analysis, reported elsewhere, we identified 6 psychoemotional items from the 26-item Health-Related Quality of Life in Stroke Patients (HRQOLISP-26), a stroke-specific measure developed from a large cross-cultural, transnational, patient-controlled sample and based on a comprehensive model [10]. These 6 items fitted a single dimensional model (HRQOLISP-E) with phenomenological and conceptual overlap with the depression framework in the 10th revision of the International Classification of Diseases (ICD-10) [11] and HADS-D [12]. They also demonstrated initial evidence of construct validity and internal consistency reliability. Those results provided preliminary support for further development of the HRQOLISP-E as the first stroke-specific screening tool for depression.

In the present study, we rely on the HADS-D as a meaningful criterion to investigate the properties of the HRQOLISP-E. We determined cut-off scores with the best balance of sensitivity and specificity, likelihood ratios, and posterior test probabilities.

## 2. Methods

### 2.1. Sites

We evaluated a dataset comprising baseline information collected as part of an ongoing study of an intervention to improve one-year blood pressure control among recent (<1 month) stroke survivors who were discharged from four hospitals in Nigeria. Ethical approval was obtained from the institutional review boards covering the four hospital sites: the University of Ibadan/University College Hospital joint ethics committees (which cover the World Federation of Neurorehabilitation-Blossom hospital site), Federal Medical Center, Abeokuta, and Sacred Heart Hospital. Participants provided written, informed consent before interviews were conducted.

### 2.2. Subjects

The subjects comprised consecutive adult ischaemic or haemorrhagic stroke survivors. The diagnosis of stroke was confirmed based on neuroimaging and clinical examination criteria [13].

The patients were informed about the study, and the procedure was explained to them in their home language. We excluded patients with severe communication difficulties (*N* = 34) or aphasia (*N* = 42) and those with severe conditions that could limit participation in follow-up assessments (*N* = 94). This included those with severe cognitive impairments or dementia [(Modified Community Screening Instruments for Dementia (CSID) ≤ 20)], global disability [(Modified Rankin Scale (MRS) ≥ 3)], and those with significant comorbid medical illnesses (e.g., chronic kidney disease) [14].

### 2.3. Measures

Stroke survivors meeting the study criteria underwent baseline assessments within the first month of stroke.

PSD was ascertained using the depression subscale of the Hospital Anxiety and Depression Scale (HADS-D) [12]. The HADS is one of the most widely used screening tools for PSD, and as reported in a recent systematic review of all such instruments [8], it is one of two tools with superior psychometric properties and clinical utility indices in stroke populations. As such, it could be considered a useful referent tool for the development. It includes a total of 14 items each with a score of between 0 and 3. One-half of the items are related to anxiety while the other half is specific for depression. The developers of the scale recommend a cut-off ≥ 8 for the ascertainment of depression in clinical settings. The HADS has been previously validated in Nigeria [15] where the HADS-D was found to have a sensitivity ranging 89.5–92.1% and a specificity of 86.6–91.1%. Given the acclaimed properties of the HADS-D, we used depression ascertained using the measure as a referent standard for the purpose of the present study.

The HRQOLISP-E was also independently administered within 15–20 minutes of the HADS. As previously stated, the HRQOLISP-E was empirically determined from the HRQOLISP-26, a stroke-specific measure developed from a large cross-cultural, transnational, patient-controlled sample and based on a comprehensive model [10]. The 6 items of the unidimensional scale overlap with the depression framework in the 10th revision of the International Classification of Diseases [11] and HADS-D [12]. These items also demonstrated initial evidence of construct validity and internal consistency reliability (item scale correlations > 0.8 (0.81–0.93), Cronbach's alpha = 0.939, split-half reliability = 0.899 versus 0.739 for HADS-D).

### 2.4. Other Data Collection

The following information was obtained from all participants using a standardized questionnaire: demographic data, personal history of smoking, alcohol consumption, physical activities, medical history of hypertension, diabetes, hyperlipidaemia and heart disease, the use of medications for these conditions, and family history. Information on dietary patterns was obtained using the food frequency questionnaire. The severity of stroke was ascertained using the National Institute of Health Stroke Scale and Stroke Levity Scale [16]. The average of two blood pressure (BP) measurements was recorded. Each BP measurement was obtained using an Omron HEM-907 XL 26 blood pressure monitor, and the readings were recorded according to standardized protocol provided by the manufacturers. Along with the blood pressure and pulse rates, anthropometric measurements of weight, height, waist, and hip circumferences were also undertaken. Records of other relevant risk factors for stroke were also made. This includes fasting blood sugar, lipid profile, electrocardiogram, carotid Doppler, and echocardiography.

### 2.5. Statistical Analyses

Descriptive statistics such as means and standard deviations were used to summarize quantitative variables, while frequencies and proportions were used for discrete variables. All analyses were conducted using Stata MP version 14.0 [17]. Values of *p* < 0.05 were considered significant.

### 2.6. Background Factor Analyses

The methods, results, and interpretation of the initial factor analyses leading to the present study are reported elsewhere. Briefly, we conducted exploratory factor analysis (EFA) on all 7 items in the HRQOLISP-26 psychoemotional domain. Factors obtained following initial maximum likelihood exploration were further rotated using the varimax procedure. Factors were recorded when they have eigenvalues greater than unity. For the factor extraction, loadings of ≥0.5 were considered meaningful.

The background EFA generated a single dimensional model (HRQOLISP-E) with phenomenological and conceptual overlap with the depression framework in the fourth revision of the diagnostic and statistical manual of mental disorders (DSM IV) [18] and HADS-D [12]. HRQOLISP-E contains items corresponding to depressed mood (2 items: *seldom/never able to laugh and dissatisfied with feelings*), loss of interest/anhedonia (2 items: regarding *work* and *leisure*), decreased energy or fatigability (1 item), and low self-esteem/confidence (1 item: *seldom/never* able to accept bodily appearance).

### 2.7. The Present Psychometric Analyses

For the present study, we compared the phenomena of being positive for depression using the HADS-D criterion versus being positive using the HRQOLISP-E. For this, we first classified the entire sample of 387 patients into four groups using the result of both measures. We determined depressed subjects in the HADS-D (total abnormal) and those showing negative results (total normal). We next determine depression-positive (true positive) and negative participants using the HRQOLISP-E (false negative) among the “total abnormal” group. Among participants belonging in the “total normal” group, we determined “true negative” when participants show depression-negative on the HRQOLISP-E and “false positive” when they show depression-positive results using the same screening.

Next, we calculated sensitivity (number of “true positive” participants divided by the number of participants in the “total abnormal” group) and specificity (number of “true negative” participants divided by the number of participants in the “total normal” groups). We also estimated likelihood ratios (LR) for positive and negative depression screen on the HRQOLISP-E and plotted these values against the proportion of “total abnormal” in the sample (pretest probability) to determine the posterior test probabilities of depression-positive screen when using the HRQOLISP-E. The Bayesian plot of the LR, pretest, and posterior test probabilities is presented.

The sensitivity and specificity values for different possible cut-off scores for defining depression in the HRQOLISP-E were also plotted on an ROC curve. The area under the curve (AUC), as well as the most appropriate HRQOLISP-E cut-off, was calculated. This cut-off value was established as the one with higher results for the sum of sensitivity and specificity.

## 3. Results

There were 248 males and 139 females in the study sample (Table 1). Their mean age was 57.4 (±11.6) years. There was no significant difference in the mean age for men (57.4 ± 12.2) and women (57.0 ± 10.8). Over 90% of the subject had at least 6 years of formal education. Nearly all participants in the present study had either mild or moderate stroke.

**Figure 1:**
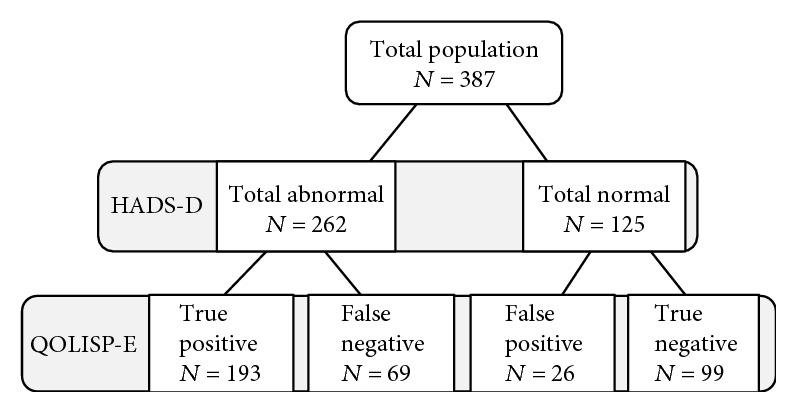
Number of patients with or without criterion-diagnosed depression who were screened depressed using the HRQOLISP-E.

Using HADS-D cutoff score of ≥8 as a criterion, depression was found in 262 (67.7%) participants (Figure 1). In the same figure, the number of patients with and without HADS-assessed depression who were screened depressed using the HRQOLISP-E is also presented.

**Figure 2:**
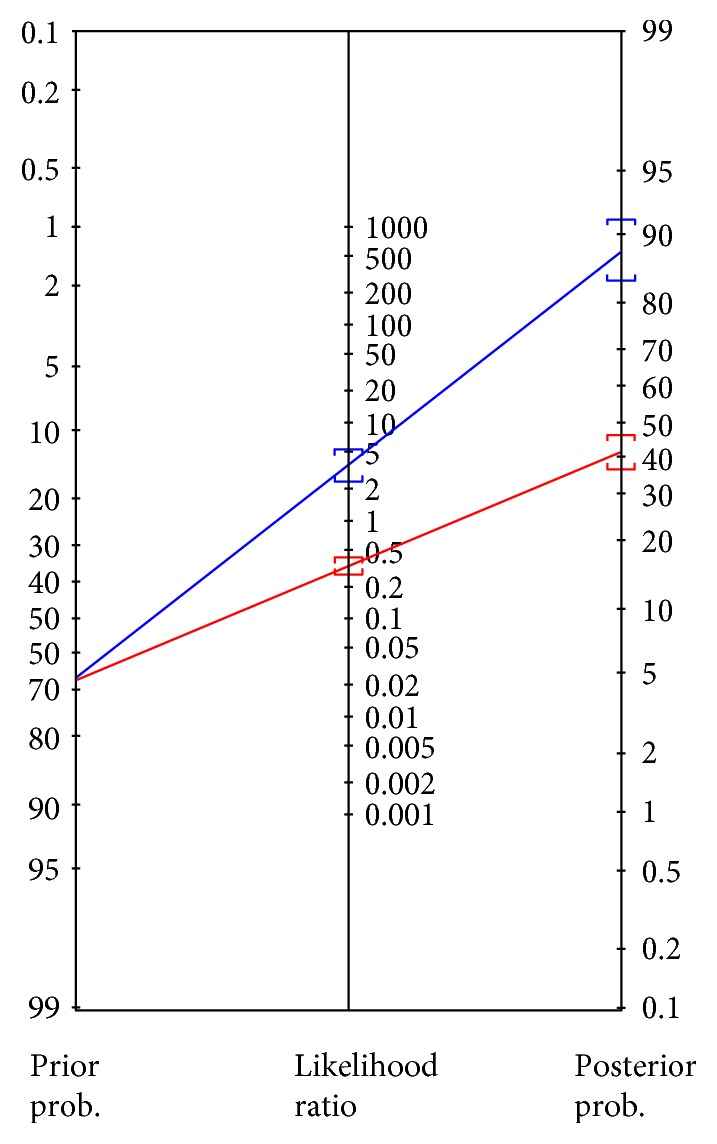
Probability that a patient is criterion-depressed after screening depressed using the HRQOLISP-E. Depression-positive HRQOLISP-E: positive likelihood ratio = 3.56 (95% CI = 2.51–5.06) and posterior test probability = 0.88 (0.84–0.91). Depression-negative HRQOLISP-E: negative likelihood ratio = 0.33 (95% CI = 0.27–0.41) and posterior test probability = 0.41 (0.36–0.46).

The items in the HRQOLISP-E determined using EFA and their scoring is presented in Table 2. The HRQOLISP-E items showed high agreement with the referent-standard HADS-D cutoff ≥ 8 with a sensitivity rate of 73.7% and specificity of 79.3. The result of the Bayesian nomogram plot indicates a posterior test probability of 88% (95% CI = 84%–91%) when using the HRQOLISP-E as *a screening tool* (Figure 2). We note that the HRQOLISP-E loses sensitivity for PSD at HADS-D criterion > 8 (sensitivity = 39.3%, specificity = 93.4%), thus making it unsuitable for definitive diagnosis of PSD.

**Figure 3:**
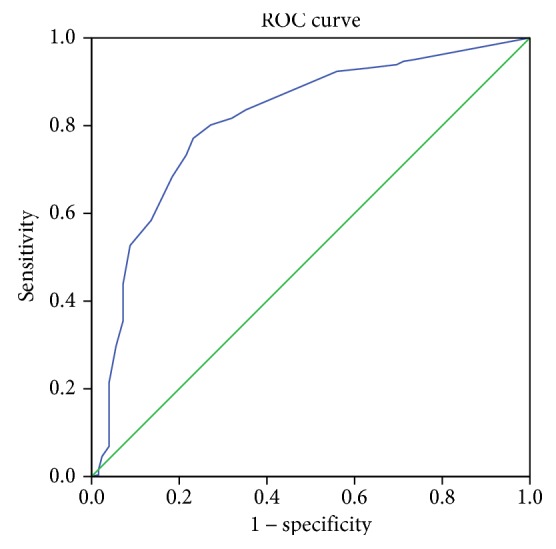
Receiver operator curve for HRQOLISP-E. Diagonal segments are produced by ties. Area under the curve = 0.81 (95% CI = 0.76–0.86), best depression screen cut-off score = 20/25, sensitivity = 77.1%, and specificity = 76.8%.

The AUC was 0.812 (95% CI = 0.764–0.859) (Figure 3). The cut-off for HRQOLISP-E depression corresponding to HDS-D ≥ 8 was 20/21, sensitivity = 77.1%, specificity = 76.6%, and a posterior test probability of 87% (95% CI = 83%–91%), with lower HRQOLISP-E scores representing more depression symptoms.

## 4. Discussion

We found in the present study that within existing data from a fairly large sample of stroke survivors, 6 empirically determined psychoemotional domain items of the HRQOLISP-26, a stroke-specific measure, showed high agreement with the HADS-D. These results would suggest that the new measure (“HRQOLISP-E”) may be useful as a stroke-specific *screening tool* for depression. If developed further, the HRQOLISP-E will be useful for rapid screening of depression in busy stroke clinics, and also in research, to determine stroke survivors who may or may not require additional clinical diagnostic assessments.

Even though routine screening of PSD is now currently recommended [4], observations from the two available systematic review of all tools that have been used for depression screening in the stroke population (*N* = 27) suggest that there are currently no verbally self-reported (i.e., not incorporating visual aids) PSD-screening tools designed with stroke specificity [8]. Available screening tools for PSD are generic and originally designed for use in general psychiatric populations [9]. As such, many tools include depression symptoms which overlap with those of stroke. However, as the experience of depression may vary across socioeconomic and clinical circumstances [19], the inclusion of symptoms with substantial overlaps with those of stroke in many commonly used screening tools for PSD may conflate scores and lead to inaccurate clinical decisions or research findings. The “HRQOLISP-E,” which is empirically designed from the HRQOLISP-26, a stroke-specific measure developed from a large cross-cultural, transnational, patient-controlled sample and based on a comprehensive model [10], may serve to fill the current gap created by the unavailability of a context-specific measure for PSD.

Another significance of the results of this study is that it provides additional advantage, in stroke studies, of using the parent HRQOLISP-26 (a multidomain stroke-specific measure of quality of life). This is because its use in the setting of stroke precludes the need for protocol inclusion of additional screening tools for depression, especially as quality of life is often also measured in such studies. Many clinical diagnostic conventions require the exclusion of important mimics of specific neurobehavioural syndromes in other to improve diagnostic precision [20]. For example, for a confident diagnosis of specific anxiety disorders, many studies may seek to exclude comorbid depression [21]. In this way, measures are often combined in studies to cover all relevant dimensions. This procedure potentially imposes additional encumbrances on stroke survivors who may already be suffering under the weight of physical and cognitive disability [22]. This situation may reduce the overall responsiveness in studies requiring such additional protocol inclusion and therefore the reliability of their findings.

If used to preclude protocol inclusion of additional screening tool for depression, we recommend that the first item in the original HRQOLISP-26 psychoemotional subscale be excluded as it showed low factor loading, and its inclusion reduced the construct validity as a depression measure in our previous investigation.

The standard recommendation for a diagnosis of PSD suggests that depression diagnoses should most appropriately be based on a semistructured mental state examination and clinical criteria such as the DSM IV/V or ICD-10 for depression due to stroke with major depressive-like episode or depressive features [23]. Given this standard recommendation, we note that the HADS-D, the criterion measure for the present study, is not the gold standard for depression ascertainment and diagnoses. We are thus mindful of the effect this particular limitation on the results of the present study. It is feasible that HRQOLISP-E may perform differently against a stronger depression criterion. However, as we have not carried out clinical diagnostic assessments as part of the present study, we have chosen the HADS-D as the next best criterion to compare HRQOLISP-E by relying on evidence from available systematic reviews and meta-analysis [8, 9].

Another limitation of the present study is that participants were identified as part of a randomized controlled trial (RCT). Persons who were too ill to provide subsequent follow-up information were excluded, thus, suggesting that the sample for the present study may not be typical of the full spectrum of stroke survivor population in the study setting.

## 5. Conclusion

The results of the present study provide preliminary support for further development of the HRQOLISP-E as a stroke-specific screening tool for depression through an investigation comparing the proposed measure against referent standard clinical diagnostic criteria such as the DSM IV/V and ICD-10. The clinical utility of screening tools for PSD will be improved if such measures reflect the user context since the experience of depression may vary across socioeconomic and clinical circumstances [19]. The “HRQOLISP-E” is empirically designed from a stroke-specific measure and appears to demonstrate high agreement with the HADS-D. Indeed, our initial reliability information shows that the new instrument may be potentially more reliable for depression screening in acute stroke compared with the HADS-D, which is not a stroke-specific tool. The findings of this study require confirmation from studies using a more generalizable sample of stroke survivors.

## Figures and Tables

**Table 1:** Characteristics of the study sample using the HADS-D criterion.

Variables	Overall sample (*N* = 387)	No depression (*N* = 125)	Depression (*N* = 262)
*Mean age*	57.4 (11.6)	55.4 (10.8)	58.3 (11.9)
*Age (years)*			
≤45	58 (15.0)	25 (20.0)	33 (12.6)
45–65	235 (60.7)	79 (63.2)	156 (59.5)
>65	94 (24.3)	21 (16.8)	73 (27.9)
*Gender*			
Male	248 (64.1)	84 (67.2)	164 (62.6)
Female	139 (35.9)	41 (32.8)	98 (37.4)
*Education*			
None	32 (8.3)	12 (9.6)	20 (7.6)
Primary	72 (18.6)	17 (13.6)	55 (21.0)
Secondary	107 (27.7)	35 (28.0)	72 (27.5)
Higher	176 (45.5)	61 (48.8)	115 (43.9)
*Occupation*			
Skilled/professional	117 (30.2)	43 (34.4)	74 (28.2)
Semiskilled	52 (13.4)	13 (10.4)	39 (14.9)
Manual	108 (27.9)	36 (28.8)	72 (27.5)
Retired	88 (22.7)	25 (20.0)	63 (24.1)
Others	22 (5.7)	8 (6.4)	14 (5.3)
*Stroke severity: NIHSS*			
Mild (≤15)	385 (99.5)	124 (99.2)	261 (99.6)
Moderate (16–20)	2 (0.5)	1 (0.8)	1 (0.4)
Severe (21–42)	—	—	—
*Stroke severity: SLS*			
Mild (11–15)	312 (81.5)	112 (91.1)	200 (76.9)
Moderate (6–10)	65 (17.0)	9 (7.3)	56 (21.5)
Severe (0–5)	6 (1.5)	2 (1.6)	4 (1.5)
*Total*	**100**	**32.3**	**67.7**

**Table 2:** Items and their scoring in the conceptually defined “HRQOLISP-E.”

HRQOLISP-E	Scoring	
(1) ^∗^*Do you have enough energy for everyday life?*	*Not at all/never* ^∗^	**1**
	A little/seldom^∗^	**2**
	Moderately/quite often	3
	Mostly/very often	4
	Completely always	5

(2) *To what extent are you able to accept your bodily image?*	*Not at all/never*	**1**
	*A little/seldom*	**2**
	Moderately/quite often	3
	Mostly/very often	4
	Completely always	5

(3) ^∗^To what extent do you enjoy your work?	*Not at all/never* ^∗^	**1**
	*A little/seldom* ^∗^	**2**
	Moderately/quite often	3
	Mostly/very often	4
	Completely always	5

(4) How often do you laugh?	*Not at all/never*	**1**
	*A little/seldom*	**2**
	Moderately/quite often	3
	Mostly/very often	4
	Completely always	5

(5) ^∗^To what extent do you enjoy leisure?	*Not at all/never* ^∗^	**1**
	*A little/seldom* ^∗^	**2**
	Moderately/quite often	3
	Mostly/very often	4
	Completely always	5

(6) How satisfied are you with your feelings	*Not at all/never*	**1**
	*A little/seldom*	**2**
	Moderately/quite often	3
	Mostly/very often	4
	Completely always	5

^∗^Equivalent to core symptom of depression in the 10th revision of the International Classification of Diseases (ICD-10). *Note*. Endorsing ≥4 shaded responses, which must include 2 core symptoms, corresponds to the conceptual framework of depression in the ICD-10.

## References

[1] Benjamin E. J., Blaha M. J., Chiuve S. E. (2017). Heart disease and stroke statistics—2017 update: a report from the American Heart Association. *Circulation*.

[2] Ojagbemi A. (2014). The high rate of major depression after stroke in Nigeria may be the result of high cumulative morbidity burden: a call for greater efficiency in the management of stroke in developing countries. *International Journal of Stroke*.

[3] Hart S., Morris R. (2008). Screening for depression after stroke: an exploration of professionals’ compliance with guidelines. *Clinical Rehabilitation*.

[4] Towfighi A., Ovbiagele B., El Husseini N. (2016). Poststroke depression: a scientific statement for healthcare professionals from the American Heart Association/American Stroke Association. *Stroke*.

[5] Mitchell P. H., Veith R. C., Becker K. J. (2009). Brief psychosocial-behavioral intervention with antidepressant reduces poststroke depression significantly more than usual care with antidepressant: living well with stroke: randomized, controlled trial. *Stroke*.

[6] National Audit Office (2010). *Progress in Improving Stroke Care*.

[7] Vuletic V., Sapina L., Lozert M., Lezaić Z., Morović S. (2012). Anxiety and depressive symptoms in acute ischemic stroke. *Acta Clinica Croatica*.

[8] Burton L. J., Tyson S. (2015). Screening for mood disorders after stroke: a systematic review of psychometric properties and clinical utility. *Psychological Medicine*.

[9] Meader N., Moe-Byrne T., Llewellyn A., Mitchell A. J. (2014). Screening for poststroke major depression: a meta-analysis of diagnostic validity studies. *Journal of Neurology, Neurosurgery, and Psychiatry*.

[10] Owolabi M. O. (2009). Health related quality of life (HRQOL) and the seed of life model. *Journal of Alternative Medicine Research*.

[11] World Health Organisation (1992). *International Classification of Diseases: 10th revision (ICD 10)*.

[12] Zigmond A. S., Snaith R. P. (1983). The hospital anxiety and depression scale. *Acta Psychiatrica Scandinavica*.

[13] Sacco R. L., Kasner S. E., Broderick J. P. (2013). An updated definition of stroke for the 21st century: a statement for healthcare professionals from the American Heart Association/American Stroke Association. *Stroke*.

[14] Owolabi M. O., Akinyemi R. O., Gebregziabher M. (2014). Randomized controlled trial of a multipronged intervention to improve blood pressure control among stroke survivors in Nigeria. *International Journal of Stroke*.

[15] Abiodun O. A. (1994). A validity study of the hospital anxiety and depression scale in general hospital units and a community sample in Nigeria. *The British Journal of Psychiatry*.

[16] Owolabi M. O., Platz T. (2008). Proposing the stroke levity scale: a valid, reliable, simple, and time-saving measure of stroke severity. *European Journal of Neurology*.

[17] StataCorp (2013). *Stata Statistical Software: Release 13*.

[18] American Psychiatric Association (1994). *Diagnostic and Statistical Manual of Mental disorders: (DSM IV) Text Revision*.

[19] Gainotti G., Azzoni A., Marra C. (1999). Frequency, phenomenology and anatomical-clinical correlates of major post-stroke depression. *The British Journal of Psychiatry*.

[20] American Psychiatric Association (2000). Delirium, dementia and amnestic and other cognitive disorders. *Diagnostic and Statistical Manual of Mental disorders, (DSM IV) Text Revision*.

[21] Kessler R. C., Sampson N. A., Berglund P. (2015). Anxious and non-anxious major depressive disorder in the World Health Organization World Mental Health Surveys. *Epidemiology and Psychiatric Sciences*.

[22] D'Olhaberriague L., Litvan I., Mitsias P., Mansbach H. H. (1996). A reappraisal of reliability and validity studies in stroke. *Stroke*.

[23] Robinson R. G. (2003). Poststroke depression: prevalence, diagnosis, treatment, and disease progression. *Biological Psychiatry*.

